# MicroRNA Is a Major Regulator

**DOI:** 10.1371/journal.pbio.0020396

**Published:** 2004-10-05

Since their discovery a decade ago, microRNAs (miRNAs) have emerged as major regulators of gene expression in eukaryotes of all kinds. Only 20 to 40 nucleotides long, a miRNA binds to a specific target sequence within a much longer messenger RNA (mRNA), inhibiting its translation and thus controlling expression of the corresponding gene even after the DNA itself has been read. Within the human genome, there are about 250 genes that code for miRNAs. Each miRNA has the potential to bind to many different transcripts. Variations in miRNA sequence dictate the gene transcripts to which each will bind most strongly.

It has become clear that miRNAs play a critical role in controlling gene expression, for example, in larval developmental transitions and neuronal development in the worm Caenorhabditis elegans, growth control and apoptosis in the fruitfly Drosophila melanogaster, hematopoietic differentiation in mammals, and leaf development, flower development, and embryogenesis in the plant Arabidopsis thaliana. Despite their significance, the full range of genes miRNAs target is unknown, as is the best method for discovering them. In a new study, Debora Marks, Chris Sander, and colleagues describe an algorithm for determining the targets of miRNAs, and show they include more than 10% of all human genes.

The algorithm uses three factors to evaluate whether a potential target site is likely to actually be regulated by miRNA. First, the target site must have some degree of sequence complementarity to one or more of the known miRNAs. Second, the strength with which the predicted target and its miRNA bind together, which can be calculated from the sequence and other structural factors, must be higher than some threshold. Finally, evolutionary conservation—the presence of the target–miRNA pair in different organisms—is factored in, because the likelihood that the target and miRNA actually pair in vivo is greater if the pair is found in multiple types of organisms.

Using these principles, and the specific weighting they assigned to each factor, Marks and colleagues identified 2,273 genes in humans, rats, and mice that are likely targets for miRNA regulation. This is probably an underestimate of the total, since the researchers required each candidate gene to have at least two miRNA target sites. The authors identified another 2,128 genes with only one target site, but note that the false-positive rate here is likely to be high. Whatever the final number, the implication is that several thousand of our approximately 30,000 genes are under the control of miRNAs. Of special interest is that these putative targets include many genes known to be associated with the fragile X mental retardation protein, a crucial but still poorly understood player in mRNA regulation, whose absence leads to a type of mental retardation called fragile X syndrome.

**Figure:**
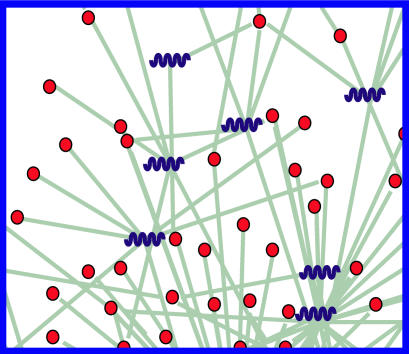
microRNA gene networks

The researchers' findings also reinforce several emerging principles of miRNA-based regulation. First, it is widespread among multicellular eukaryotes, and sequences are surprisingly conserved. Of the 78 known miRNAs in Drosophila, 28 have close relations in mammals. Second, an individual miRNA may regulate multiple genes—Marks and colleagues found that the average miRNA interacts with seven distinct mRNAs, with a range from 0 to 268. Third, the genes regulated by a single miRNA may be functionally related, such as components of the protein degradation system or specific signal transduction pathways. Fourth, single genes may be regulated by multiple miRNAs—the gene that encodes amyloid precursor protein, for example, has at least eight miRNA sites—suggesting that expression may be combinatorially controlled by numerous cellular influences.

These results provide resources for a host of experiments to elucidate the mechanism of miRNA action, which is not well understood. Several of the identified mammalian miRNA–target pairs have near-perfect matching sequences. In both plants (where miRNAs were first discovered) and animals, such matches are associated with degradation of the mRNA.

The authors fully recognize that their algorithm, called miRanda, is not the last word in miRNA target identification. In order to improve both the search for targets and the algorithm itself, they are making the algorithm and full sets of results in vertebrates available free to other researchers (www.microrna.org), who can modify its parameters as experimental results and new models dictate.

